# Supplementary material for: Content-rich biological network constructed by mining PubMed abstracts
Source: BMC Bioinformatics. 2004 Oct 8;5:147. doi: 10.1186/1471-2105-5-147 (PMC528731; doi:10.1186/1471-2105-5-147)
Supplement: Additional File 2 — The original results of the above study (non-essential files are deleted to keep the file size under the limit set by BMC bioinformatics). [file 1471-2105-5-147-S2.bz2 › chilibotAdditionalFile2/dip40/96ID9324938E403/html/BAD_BCL2L1.html]

 


 **BAD** and **BCL2L1** 
  
Found 251 abstracts in PubMed, retrieved 40.  
 

 What does Google say? 
 PDF only 
| .edu only 

---

**Interactive relationship** (e.g. stimulation, inhibition, etc)

**Stimulatory relationship**- In WtFLT3 transfected 32D WtFLT3 32D cells, FLT3 ligand FL stimulation did not restore the down regulation of Bcl XL  [ **BCL2L1** ]  but maintained the phosphorylation of  **Bad** .  Ref: 12842996 Blood< MedlineTA>Blood,
- Similarly, a peptide derived from the BH3 domain of  **Bad**  stimulates Bax activity only in the presence of Bcl xL  [ **BCL2L1** ] .  Ref: 12642586 J Biol Chem, 2003
- Moreover TIMP 1 enhances specific phosphorylation of both Akt and  **Bad**  in a PI 3 kinase dependent manner and besides controls the level of the anti apoptotic protein Bcl X  [ **BCL2L1** ]  L.  Ref: 12639219 Biochem J< MedlineTA>Biochem J, 2003
**Neutral relationship**- We describe the implementation of confocal and two photon excitation fluorescence resonance energy transfer FRET microscopy techniques through which we demonstrate interaction between the proapoptotic protein  **BAD**  and the prosurvival protein Bcl xL  [ **BCL2L1** ]  within TAI following TBI.  Ref: 12880338 J Biomed Opt, 2003
- Coimmunoprecipitation revealed that the dimerization of  **Bad**  progressed with 14 3 3  **Bad**  14 3 3 and with Bcl x  [ **BCL2L1** ]  L  **Bad**  Bcl x  [ **BCL2L1** ]  L after tFCI.  Ref: 12629175 J Neurosci, 2003
- Cleavage of 14 3 3 Protein by Caspase 3 Facilitates  **Bad**  Interaction with Bcl x  [ **BCL2L1** ]  L during Apoptosis.  Ref: 12657644 J Biol Chem, 2003
- However,  **Bad**  associated with the cellular Bcl x  [ **BCL2L1** ]  L more effectively in human 293T cells co expressing  **Bad**  with the truncated form of the 14 3 3epsilon protein D238 than in control cells co expressing  **Bad**  with wild type or the uncleavable mutant 14 3 3epsilon protein D238A.  Ref: 12657644 J Biol Chem, 2003
- CD44v7 deficiency was characterized by an increase in the percentage of apoptotic cells after stimulation, increased numbers of CD95L and CD152 positive cells, low levels of the anti apoptotic proteins Bcl 2 and Bcl Xl  [ **BCL2L1** ] , and decreased phosphorylation of the pro apoptotic protein  **BAD** .  Ref: 12832452 J Leukoc Biol, 2003

**Non-interactive relationship** (e.g. studied together, co-existance, homology, etc.)

- First, the Bcl xL  [ **BCL2L1** ]   **BAD**  ratio in PC 3 cells is at least an order of magnitude greater than that of LNCaP cells.  Ref: 12738789 J Biol Chem< MedlineTA>J Biol Chem, 2003
- mRNA expression of Bcl w,  **Bad** , and Bcl x  [ **BCL2L1** ]  was not significantly different between the control and TPN groups.  Ref: 12471468 Pediatr Surg Int, 2002
- These proteins include the death antagonists Bcl 2 and Bcl X  [ **BCL2L1** ]  L, and death agonists Bax and  **Bad** .  Ref: 12507784 J Neuroimmunol, 2003
- This study examined cutaneous T cell lymphoma CTCL cell lines and cutaneous lesions for the presence of Bcl 2 gene family members and found that the two apoptosis inhibiting members Bcl xL  [ **BCL2L1** ]  and Mcl 1 and the two apoptosis supporting members  **Bad**  and Bax were expressed.  Ref: 12754746 J Pathol, 2003
- One proposed pathway by which this has been hypothesized to occur is the Ca 2 mediated activation of calmodulin and subsequent activation of the phosphatase calcineurin with dephosphorylation of a protein known as  **BAD** , leading to a proapoptotic interaction between  **BAD**  and the mitochondrial protein Bcl xL  [ **BCL2L1** ] .  Ref: 12880338 J Biomed Opt, 2003
- While this pathway is an intriguing route for traumatic axonal pathogenesis, neither conventional immunocytochemical histochemical nor ultrastructural approaches have had the capacity to shed insight on whether  **BAD**  and Bcl xL  [ **BCL2L1** ]  interact in TAI in vivo.  Ref: 12880338 J Biomed Opt, 2003
- **Bad**  Bcl x  [ **BCL2L1** ]  L was prevented by SOD1 but promoted by H89 treatment.  Ref: 12629175 J Neurosci, 2003
- Immunohistochemistry and flow cytometry were employed for detection of expressions of bcl 2, bcl xl  [ **BCL2L1** ] , bax and  **bad**  proteins.  Ref: 12658745 J Huazhong Univ Sci Technolog Med Sci, 2002
- Western blot analysis of Bcl 2, Bcl xL  [ **BCL2L1** ] ,  **Bad** , Caspase 3, PDK1, and phospho Akt also revealed SOD mediated changes in gene expression consistent with protection and decreased apoptosis.  Ref: 12668130 Mol Ther, 2003
- FLT3 kinase inhibitor, AG1296, alone not only dephosphorylated  **Bad**  but also down regulated Bcl XL  [ **BCL2L1** ] , leading FLT3 ITD 32D cells into apoptosis.  Ref: 12842996 Blood< MedlineTA>Blood, 2003
- The results of immunohistochemistry demonstrated that bcl 2, bcl xl  [ **BCL2L1** ] , bax and  **bad**  proteins were constitutionally present on the sarcolemma of the LV myocytes.  Ref: 12658745 J Huazhong Univ Sci Technolog Med Sci, 2002
- In turn,  **BAD**  dimerized with antiapoptotic BCL Xl  [ **BCL2L1** ]  after seizures.  Ref: 12728252 Cell Death Differ, 2003
- 3 Treatment with 80 micro M magnolol significantly increased the expression of  **Bad**  and Bcl X  [ **BCL2L1** ]  proteins.  Ref: 12522090 Br J Pharmacol, 2003
- Apoptosis was studied by the terminal deoxynucleotidyl transferase mediated deoxy UTP nick end labeling method, and immunohistochemistry was used to detect p53, caspase 3 and 6, the antiapoptotic proteins Bcl 2 and Bcl x  [ **BCL2L1** ] , and the proapoptotic proteins Bax and  **Bad** .  Ref: 12450319 Endocrine, 2002
- Since Bcl 2 family proteins are key regulators of apoptosis, we examined the effects of H2O2 on the expression of principal Bcl 2 family proteins Bcl 2, Bcl xL  [ **BCL2L1** ] , Bax,  **Bad**  in neonatal rat cardiac myocytes.  Ref: 12721309 J Biol Chem< MedlineTA>J Biol Chem, 2002
- Oxidative stress induced early apoptosis was linked to its ability to inhibit not only the expression of Bcl 2 and Bcl XL  [ **BCL2L1** ]  but the production of antioxidant enzymes as well and to stimulate  **Bad**  expression.  Ref: 12757172 Dig Dis Sci, 2003
- After treatment with DADAG 8 micrograms.mL 1 for various times, the Bcl XL  [ **BCL2L1** ]  protein level decreased in a time dependent manner, while the  **Bad**  protein level was upregulated.  Ref: 12567892 Yao Xue Xue Bao, 2002
- Remarkably, these events occurred in the absence of any reduction in the expression of the Bcl 2 family members Bcl 2, Mcl 1 and Bcl XL  [ **BCL2L1** ] , or any change in the proapoptotic molecules  **Bad**  or Bax.  Ref: 12842827 Am J Physiol Gastrointest Liver Physiol< MedlineTA>Am J Physiol Gastrointest Liver Physiol, 2002
- The Bcl x  [ **BCL2L1** ] , Mcl 1,  **Bad** , and Bax proteins were also expressed in all CTCL skin lesions tested.  Ref: 12754746 J Pathol, 2003
- Furthermore, the dephosphorylation of  **Bad**  using LY294002 and PD98059 was insufficient for apoptosis, and the down regulation of Bcl XL  [ **BCL2L1** ]  using antisense treatment was needed to induce apoptosis.  Ref: 12842996 Blood< MedlineTA>Blood, 2003
- In contrast to Bcl xL  [ **BCL2L1** ] , Bcl 2 expression levels are similar in both cells lines, and do not respond to serum stimulation, suggesting that Bcl 2 may not play a physiological role in antagonizing apoptosis signals pertinent to  **BAD**  activation in prostate cancer cells.t  Ref: 12738789 J Biol Chem< MedlineTA>J Biol Chem, 2003
- ... cellular FLICE like inhibitory protein long c FLIPL, Bcl XL  [ **BCL2L1** ] , Bax, and  **Bad**  expression remains unchanged.  Ref: 12731064 Eur J Immunol, 2003
- The present study suggests that the cleavage of 14 3 3 protein during apoptosis promotes cell death by releasing the associated  **Bad**  from the 14 3 3 protein and facilitates  **Bad**  translocation to the mitochondria and its interaction with Bcl x  [ **BCL2L1** ]  L.  Ref: 12657644 J Biol Chem, 2003
- To examine p75 NTR dependent apoptosis in tumor cells, we demonstrated that a dose dependent increase in p75 NTR expression was associated with a concomitant increase in the mitochondrial proapoptotic effector proteins  **Bad** , Bax and Bik and a decrease in the mitochondrial prosurvival effector proteins phospho  **Bad** , Bcl 2 and Bcl x  [ **BCL2L1** ]  L.  Ref: 12672029 Int J Cancer, 2003
- In a murine myeloid cell line, 32D, IL 3 deprivation induced apoptosis following the down regulation of Bcl XL  [ **BCL2L1** ]  and the dephosphorylation of  **Bad** .  Ref: 12842996 Blood< MedlineTA>Blood, 2003
- apoptosis related proteins Bcl 2, Bcl xl  [ **BCL2L1** ] , Bax, Bcl xs  [ **BCL2L1** ] ,  **Bad** , phosphorylated  **Bad** , phosphorylated Akt, and phosphorylated c Jun N terminal kinase.  Ref: 12711928 Alcohol Clin Exp Res, 2003
- Here, we found that HDB induced apoptotic cell death was accompanied by upregulation of cyclin D3, Bax, and p21 and down regulation of Bcl X  [ **BCL2L1** ]  L, while HDB had no effect on the levels of Bcl 2 and  **Bad**  protein.  Ref: 12822933 J Agric Food Chem< MedlineTA>J Agric Food Chem, 2003
- FACS results showed that, after CPB, expressions of bax and  **bad**  in CPB group were significantly upregulated, while the expressions of bcl 2 and bcl xl  [ **BCL2L1** ]  were not significantly changed in both groups.  Ref: 12658745 J Huazhong Univ Sci Technolog Med Sci, 2002
- Whether conventional hypothermic CPB induces myocyte apoptosis in dog hearts and modulation of bcl 2, bcl xl  [ **BCL2L1** ] , bax,  **bad** , and caspase 3 pathways in this setting was investigated.  Ref: 12658745 J Huazhong Univ Sci Technolog Med Sci, 2002
- Furthermore, the activity of the pro apoptotic protein  **Bad**  Bcl 2 Bcl X  [ **BCL2L1** ]  L antagonist, causing cell death was completely unaffected.  Ref: 12482325 Biochem J, 2003
- Furthermore, Bcl 2 family members  **Bad** , Bak, and Bcl xS  [ **BCL2L1** ]  protein levels were increased in FHIT transfected clones when compared with Panc 1 cells.  Ref: 12890991 Pancreatology, 2003
- ... other Bcl 2 family proteins including Bax, Bcl 2, Bcl XL  [ **BCL2L1** ] , and  **Bad**  remained unchanged.  Ref: 12445860 Biochem Pharmacol, 2002
- LPA had no effect on Bcl xl  [ **BCL2L1** ] ,  **Bad** , and Bak mRNA or protein expression.  Ref: 12684213 Am J Physiol Gastrointest Liver Physiol, 2003
- **Bad**  and Bcl X  [ **BCL2L1** ]  mRNA transcription increased significantly.  Ref: 12830043 J Thorac Cardiovasc Surg, 2003
